# Selective alterations in CA1 spine morphology following dietary fructose intake

**DOI:** 10.1007/s00429-026-03102-y

**Published:** 2026-05-05

**Authors:** Mátyás Kapiller, G. Mark Marcello, Diána Hazai, Emese Andrásovszky, Péter Sótonyi, József Szabó, Bence Rácz

**Affiliations:** 1 Department of Anatomy and Histology, University of Veterinary Medicine, Budapest, Hungary; 2 Institute for Animal Breeding, Nutrition and Laboratory Animal Science, István u. 2, Budapest, 1078 Hungary

**Keywords:** Fructose, Hippocampus, CA1, Synaptic Ultrastructure, Dendritic Spines, Electron Microscopy, Metabolism, Structural Plasticity

## Abstract

High fructose intake has been linked to metabolic and cognitive disturbances, yet its effects on hippocampal synaptic architecture remain unclear. We examined whether four weeks of fructose feeding alter metabolic parameters or CA1 synaptic ultrastructure in adult rats maintained on isocaloric AIN-93G diets containing fructose, glucose, or starch as the primary carbohydrate source. Serum biochemical and hormonal profiles showed only modest, diet-specific differences without major metabolic disruption. Quantitative electron microscopy revealed similar dendritic spine density, postsynaptic density length, perforated synapse frequency, and multisynaptic bouton density across groups, whereas fructose-fed rats displayed a small but significant reduction in spine area and an alteration in circularity. These localized geometric changes occurred without broader synaptic remodeling. Overall, our findings indicate that short-term fructose exposure under metabolically controlled, solid-diet conditions produces minimal metabolic and ultrastructural effects, in contrast to the pronounced disturbances reported in metabolically stressful paradigms, suggesting that structural consequences of fructose depend strongly on dietary context and metabolic load.

**Supplementary Information:**

The online version contains supplementary material available at 10.1007/s00429-026-03102-y.

## Introduction

Excessive dietary sugar intake is a defining feature of modern Western nutrition and has been increasingly implicated in metabolic dysfunction, systemic inflammation, and cognitive decline. (Ma et al. 2022; Huang et al. 2023) In processed foods, sugar is commonly present as sucrose or high-fructose corn syrup (HFCS), both of which supply substantial amounts of fructose, a monosaccharide with distinct metabolic properties compared to glucose. Unlike glucose, fructose is metabolized largely independently of insulin and primarily in the liver, a process that can promote lipogenesis, oxidative stress, and metabolic dysregulation when consumed in excess. (Alam et al. 2022) These metabolic disturbances have raised concerns regarding fructose’s potential impact on the central nervous system, particularly the hippocampus, a brain region vital for learning, memory consolidation, and behavioral regulation. Recent syntheses highlight that fructose exposure can promote neuroinflammation, mitochondrial dysfunction, oxidative stress, and insulin resistance in the brain, processes that plausibly link peripheral metabolism to impaired hippocampal plasticity and cognition. (Spagnuolo et al. 2020)

A growing body of evidence suggests that high fructose intake alters hippocampus-dependent cognition. Rodent studies have reported impaired performance in hippocampal learning paradigms following fructose supplementation, including deficits in the Morris water maze and object-context tasks, accompanied by reductions in long-term potentiation (LTP), changes in long-term depression (LTD), diminished neurogenesis, and molecular signatures of synaptic dysfunction (Cisternas et al. 2015; Hsu et al. 2015; Noble et al. 2017). Parallel findings in rats and mice indicate that fructose exposure increases inflammatory markers—such as TNF-α and GFAP—in hippocampal homogenates and reduces mitochondrial function or abundance (Cigliano et al. 2018), changes frequently interpreted as evidence for compromised synaptic integrity. At the same time, work in aging models underscores that outcomes can be heterogeneous: in aged rats, chronic 11% HFCS consumption did not adversely affect learning, memory, or hippocampal/total brain volumes, suggesting age and experimental context may critically shape detectable effects. (Çevreli et al. 2025)

However, while previous work demonstrates functional and biochemical alterations, the structural correlates of these effects remain poorly defined. The hippocampal CA1 region is among the most extensively characterized areas for synaptic ultrastructure. Dendritic spines in CA1 stratum radiatum exhibit dynamic morphological changes in response to synaptic activity; spine enlargement typically accompanies LTP, whereas spine shrinkage or simplification accompanies LTD (Harris and Stevens 1989; Harris and Weinberg 2012). Additional ultrastructural markers, such as perforated postsynaptic densities (pPSDs) and multisynaptic boutons (MSBs), reflect enhanced synaptic efficacy or robust presynaptic output, respectively (Sorra and Harris 1993; Toni et al. 1999; Bourne and Harris 2008). These structural features serve as quantifiable readouts of synaptic strength and plasticity and are therefore essential for testing whether fructose-induced cognitive dysfunction is accompanied by measurable synaptic remodeling.

Despite the strong functional evidence of fructose-induced hippocampal alterations, only limited work has examined synaptic ultrastructure directly, and findings remain inconclusive. Some studies suggest that adolescent animals may be especially vulnerable to sugar-induced cognitive impairments, whereas adult animals show modest or transient effects (Cisternas et al. 2015; Hsu et al. 2015; Noble et al. 2017). Furthermore, differences in experimental design—such as sugar concentration, delivery method (water vs. solid feed), age of the animals, and duration of exposure—complicate interpretation across studies. Dietary background also matters: high-fat–fructose paradigms can alter hippocampal synaptic plasticity and lipid profiles, emphasizing that macronutrient context interacts with fructose exposure (Micháliková et al. 2018). Fructose transport into the brain is generally attributed to GLUT5; however, regional and cellular distributions remain debated: while some reports describe GLUT5 protein in selected neuronal and glial populations, Oppelt et al. (2017) did not detect GLUT5 expression in the hippocampal neurons using their methodological approach. These findings highlight ongoing uncertainty regarding cell-type–specific fructose handling in the adult brain. Yet whether fructose’s effects manifest as quantifiable changes in synaptic morphology remains unresolved.

To address this gap, we investigated the impact of four weeks of high-fructose feeding on the ultrastructure of CA1 synapses in adult rats using quantitative transmission electron microscopy. By comparing fructose-fed animals with glucose-fed and starch-fed controls under otherwise identical dietary conditions, we aimed to determine whether fructose produces characteristic structural signatures within the neuropil that parallel functional disturbances described in previous studies. Based on reports of fructose-induced inflammation, metabolic disruption, and cognitive impairment, we hypothesized that fructose would reduce spine size, alter PSD morphology, or decrease the frequency of synaptic specialization indicators (pPSDs, MSBs). Contrary to this hypothesis, our results reveal only minimal fructose-induced ultrastructural changes, prompting reevaluation of how fructose influences hippocampal circuitry and which mechanisms underlie fructose-associated cognitive and metabolic phenotypes.

## Materials and methods

All procedures involving animals were approved by the Institutional Animal Care and Use Committee of the University of Veterinary Medicine (permit numbers MAB53/2013 and 20/2015) and adhered to the European Council Directive 86/609/EEC. Male Wistar rats (Crl: WI BR SPF), eight weeks old at the start of the experiment, were housed at 22 °C on a 12:12-hour light–dark cycle (lights on at 07:00) with LIGNOCEL S3-4 softwood bedding. The animals were checked daily, maintained under standardized environmental conditions, and provided unrestricted access to water.

The cohort used in this study represented a typical example of the 3Rs (Replacement–Reduction–Refinement) principle. All animals enrolled here were originally part of a larger nutritional study examining hepatic physiology. Importantly, none of those unrelated experiments involved any pharmacological, surgical, or environmental treatments capable of affecting the central nervous system. The animals received no interventions beyond the dietary manipulations described below, ensuring that their hippocampal structure and function were not influenced by any external procedures. Utilizing this shared cohort reduced the total number of animals required for research while preserving experimental integrity.

After a one-week acclimatization period on a semi-synthetic control diet, rats were randomly assigned to three dietary treatment groups, each consisting of eight animals (*n* = 7 or 8 per group). The diets followed AIN-93G formulation guidelines (Reeves et al. 1993) and differed exclusively in the carbohydrate fraction, which accounted for 65% of total diet weight. In the fructose group, starch was replaced by fructose; in the glucose group, by glucose; while the control group continued to receive starch. All other dietary components were identical across groups (Tables 1 and 2). The feeding period lasted four weeks, during which all diets were provided *ad libitum.* Body weight was monitored weekly. Because the diets were isocaloric and compositionally controlled, observed differences could be attributed specifically to the carbohydrate source. Food intake was not recorded on an individual or cage basis during the experimental period. All animals were housed under identical environmental conditions and had *ad libitum* access to their assigned semi‑synthetic diets, but quantitative intake data were not collected.

To evaluate systemic metabolic responses, blood samples were collected at the time of perfusion. Serum glucose, total cholesterol, triglycerides, fructosamine, and lactate dehydrogenase activity were measured photometrically using an Olympus 400 wet chemistry analyzer at the university’s Physiology and Diagnostics Laboratory. Concentrations of insulin, glucagon, and leptin were determined using rat-specific ELISA kits (Sigma RAB0335 for leptin, Sigma RAB0904 for insulin, and RayBioTech P06883 for glucagon) on a Roche Cobas e 411 immunochemistry system (DRC Kft., Balatonfüred, Hungary). For visualization, distribution‑based plots (violins with jittered datapoints) were generated from Gaussian resampling of group means, SD, and n (visualization only). We overlaid mean (wide tick) and mean ± SE (narrow ticks) for each group. All inferential statistics were computed from the original summary‑level metrics using Welch tests with Bonferroni correction. For serum variables, violin plots with jittered datapoints were generated from Gaussian resampling based on group mean, SD, and *n* for visualization only; all inference used Welch tests on summary metrics. (Fig. 3.)

**Table 1:** Composition of the feeds

Casein	%	10	10	10
Milk protein isolate	%	10	10	10
Corn oil	%	5	5	5
Cellulose	%	5	5	5
AIN-93 mineral mixture	%	3.5	3.5	3.5
Vitamin AIN-93 blend	%	1	1	1
Cystine supplement	%	0.3	0.3	0.3
Choline chloride supplement	%	0.2	0.2	0.2
**Glucose**	%	65		
**Fructose**	%		65	
**Corn Starch**	%			65
Total	%	100	100	100

**Table 2:** Measured content of the feeds

	Dry matter	Crude ash	Crude fibre	Crude protein	Crude fat	*N*-free extractable matter
	%	in % of dry matter				
Glucose group	95.6	3.74	5.23	17.68	5.05	68.29
Fructose group	97.8	3.44	5.11	17.48	5.02	68.95
Starch group	94.5	4.24	5.40	17.85	4.98	67.53

At the end of the dietary intervention, rats were anesthetized with pentobarbital sodium (Eutanyl; 0.5 ml/kg) and perfused transcardially with 4% paraformaldehyde and 0.2% glutaraldehyde in 0.1 M phosphate buffer (pH 7.4). Brains were removed, post-fixed overnight at 4 °C, and sectioned coronally at 60 μm using a Leica VT1000 vibratome. No macroscopic differences in hippocampal size or morphology were noted across diet groups.

For electron microscopy, sections containing dorsal hippocampus (approximately − 4.0 mm from Bregma) were post-fixed in 1% osmium tetroxide, stained with 1% uranyl acetate, dehydrated through graded ethanol, and embedded in Durcupan ACM epoxy resin. Small trapezoids of CA1 stratum radiatum were cut and mounted on resin blocks. Ultrathin sections were made with a Reichert ultramicrotome, mounted on 300-mesh copper slot grids, stained with lead citrate, and examined using a JEM-1011 transmission electron microscope equipped with a Mega-View III digital camera. All micrographs were acquired at a magnification of 20,000×, and sampling locations within the neuropil were chosen in a pseudo-random manner. This single-section sampling approach, applied consistently across groups, follows established quantitative protocols and provides reliable between-group comparisons while minimizing sampling error (Babits et al. 2016).

**Fig. 1:**
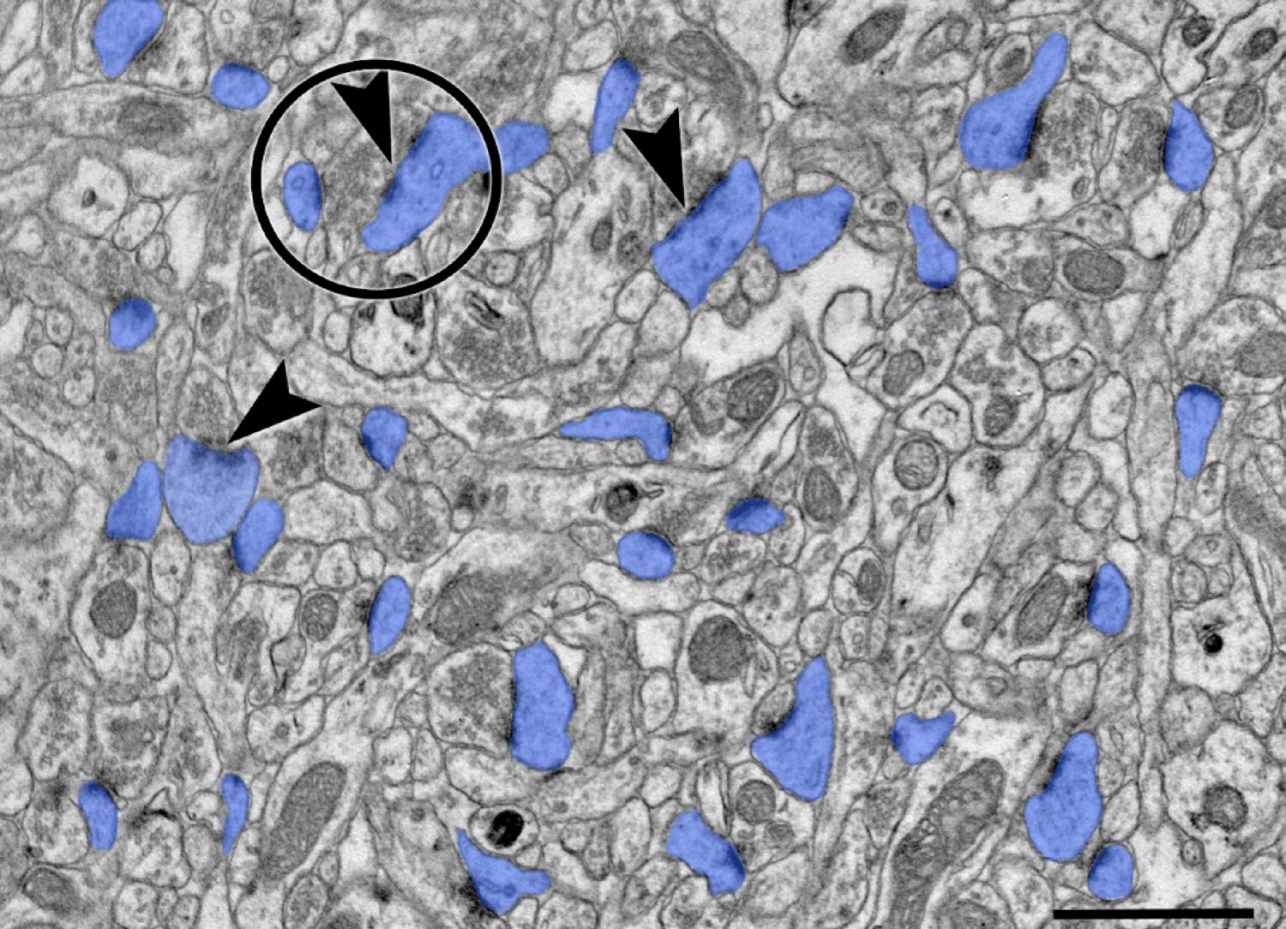
Ultrastructural analysis of the CA1 stratum radiatum neuropil. Electron micrograph at 20,000X magnification; blue pseudocolor show clearly identified synsptic spines from which spine profile perimeter, area, andcircularity was measured and calculated with ImageJ; circle show the appearance of an MSB and a perforated PSD. pPSDs are indicated with arrow. Scale bar: 1000 nm

Ultrastructural analyses were performed using NIH ImageJ software. Within defined neuropil areas of 100 μm², dendritic spine density was determined by counting identifiable spine profiles. For each spine, the profile area, perimeter, and circularity were measured, with circularity calculated as 4π·area/perimeter² (Fig. 1.) Postsynaptic density (PSD) length was measured along the electron-dense postsynaptic membrane specialization. Perforated PSDs were identified by the presence of discontinuities in the postsynaptic density, and multisynaptic boutons (MSBs) were defined as single axonal boutons forming distinct synapses with at least two dendritic spines. Quantification was conducted by an investigator blinded to experimental group to avoid bias.

Ultrastructural variables were analyzed independently using all available numeric measurements within each diet group and compared using the same Welch‑based approach, with a significance threshold of *P* < 0.05 (adjusted). For visualization of ultrastructural data, violin plots with jittered datapoints were generated directly from the raw measurements; no synthetic datapoints were used. Plots include mean and SE tick markers. Data are presented as mean ± SE unless otherwise indicated. Representative CA1 stratum radiatum electron micrographs for each dietary group are provided in Supplementary Fig. 1. for reference.

## Results

### Body weight and general physiological parameters

**Fig. 2:**
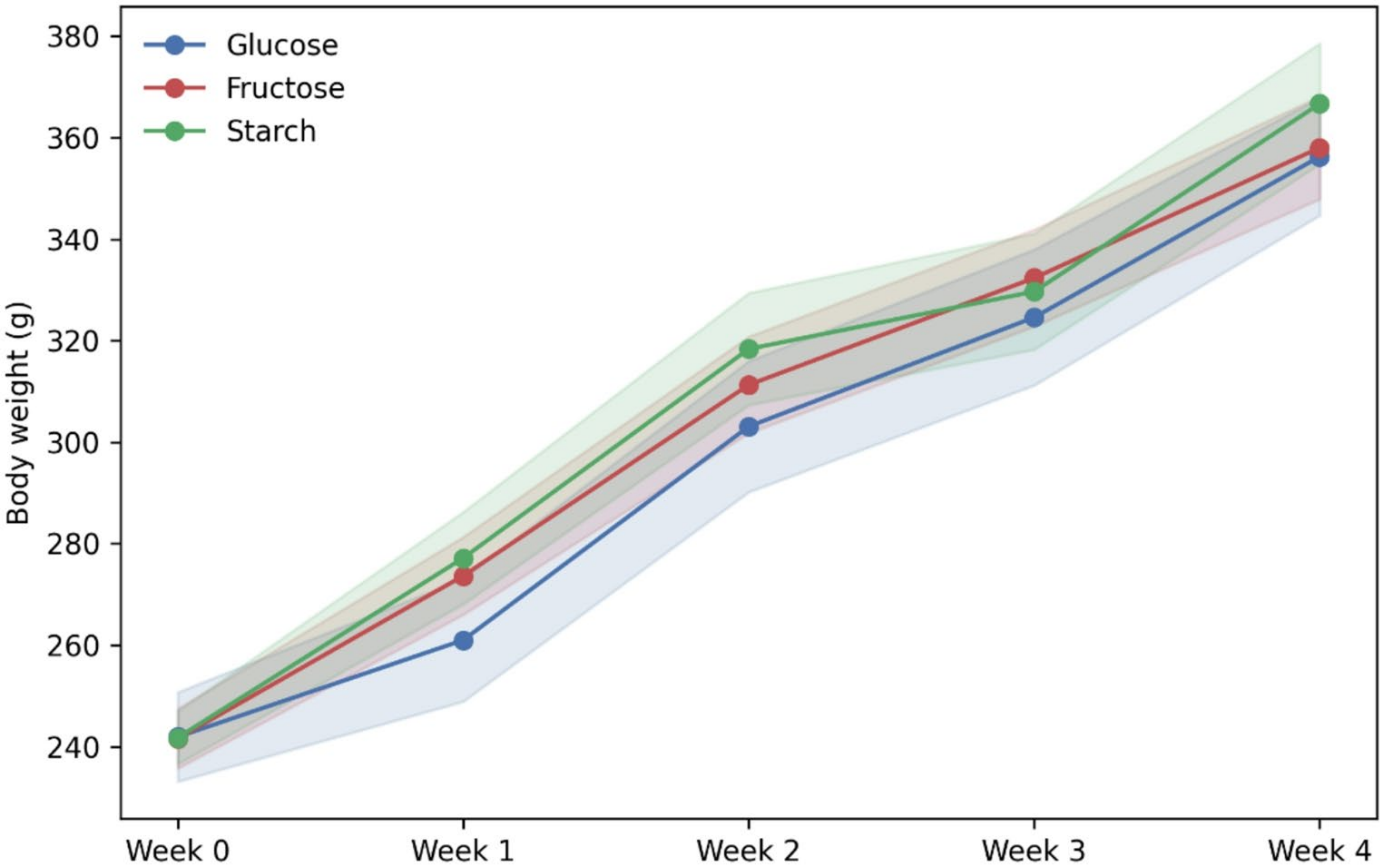
Body weight changes during the four-week dietary intervention. Weekly body weights of rats fed fructose-, glucose-, or starch-based isocaloric AIN-93G diets. All groups showed progressive weight gain across the intervention period, with no between-group differences at any time point; mean ± SE shown (*n* = 8 per group)

All animals tolerated the four-week dietary intervention well, and no abnormalities in behavior or general health were observed during the study. Body weight increased progressively in all groups, and no between-group differences were detected at any time point under the isocaloric solid-diet conditions. The fructose group exhibited a body-weight trajectory closely matching that of the glucose group, and neither monosaccharide-fed group differed significantly from the starch-fed controls at any time point. These findings indicate that, under the present feeding conditions, fructose and glucose exerted comparable orexigenic effects and did not produce differential changes in somatic growth (Fig. 2).

**Fig. 3:**
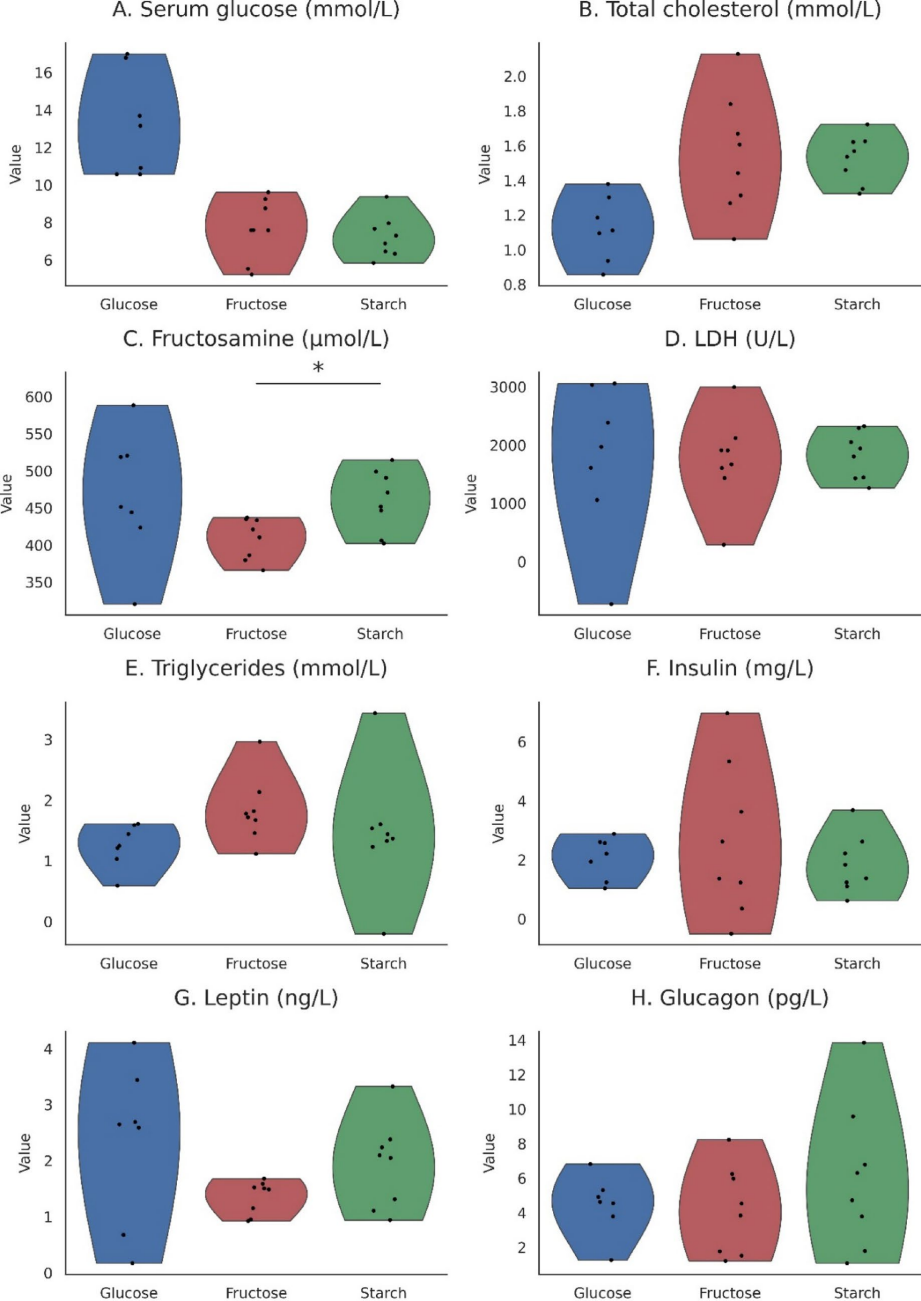
Serum biochemical parameters and hormones. (A) Serum glucose (mmol/L). (B) Total cholesterol (mmol/L). (C) Fructosamine (µmol/L). (D) LDH activity (U/L). (E) Triglycerides (mmol/L). (F) Insulin (mg/L). (G) Leptin (ng/L). (H) Glucagon (pg/L)

**Serum biochemical analyses** revealed selective, diet‑dependent differences in glycaemic indices, whereas lipid markers and peptide hormones remained broadly comparable across groups. Serum biochemical parameters are summarized in Table 3 and visualized in Fig. 3. Based on summary‑data Welch tests applied to the pooled mean ± SD values, serum glucose tended to be higher in glucose‑fed rats (11.40 ± 3.54 mmol/L, *n* = 7) relative to starch‑fed controls (7.58 ± 1.23 mmol/L, *n* = 8; *p* = 0.029), although this contrast did not remain significant after Bonferroni correction for the three pairwise comparisons (P_adj_ = 0.087). Glucose versus fructose (8.36 ± 1.64 mmol/L, *n* = 8) showed a nonsignificant trend (*p* = 0.070; P_adj_ = 0.210). Starch and fructose did not differ (*P* = 0.301). These patterns indicate a modest elevation in circulating glucose associated with glucose‑based feeding, though not statistically robust after correction.

In contrast, fructosamine levels showed a clear diet‑related shift: fructose‑fed animals had significantly lower fructosamine (398 ± 38 µmol/L, *n* = 8) than both glucose‑fed (488 ± 95 µmol/L, *n* = 7; *p* = 0.048, P_adj j_ = 0.144) and starch‑fed rats (455 ± 44 µmol/L, *n* = 8; *p* = 0.015, P_adj_ = 0.045). The fructose–starch contrast remained significant after Bonferroni adjustment, indicating that short‑term fructose feeding led to a measurable reduction in protein‑bound glycation markers under the present isocaloric, solid‑diet conditions. Serum total cholesterol showed nominal differences across groups (glucose = 1.27 ± 0.29 mmol/L; fructose = 1.61 ± 0.28 mmol/L; starch = 1.59 ± 0.18 mmol/L), with glucose lower than both fructose (*p* = 0.039) and starch (*p* = 0.031) at the uncorrected level; however, neither comparison survived Bonferroni correction (P_adj_ > 0.09), indicating no statistically robust group difference. Other circulating markers—including LDH activity (glucose = 1643 ± 905 U/L; fructose = 1842 ± 781 U/L; starch = 1747 ± 596 U/L), triglycerides, insulin, leptin, and glucagon—showed no significant pairwise differences (all *P* ≥ 0.15), with Bonferroni‑adjusted values all nonsignificant.

Together, these findings demonstrate that dietary carbohydrate type produced modest, selective effects on indices of glycaemic control—most notably a reduction in fructosamine in fructose‑fed animals—while lipid profiles, LDH activity, and peptide hormone concentrations remained stable across diets in this four‑week intervention.

**Table 3:** Serum biochemistry and hormone levels across diet groups. Group means ± SD (with sample size n) and corresponding standard errors (SE) are shown for all serum biochemical and hormonal measurements. Pairwise group comparisons were performed using Welch’s t-tests with Bonferroni-adjusted p-values (Padj) reported in the final column.

Marker	Glucose (Mean ± SD; *n*)	Fructose (Mean ± SD; *n*)	Starch (Mean ± SD; *n*)	SE (G)	SE (F)	SE (S)	Pairwise Welch tests (Bonferroni-adjusted)
Serum glucose (mmol/L)	11.40 ± 3.54 (7)	8.36 ± 1.64 (8)	7.58 ± 1.23 (8)	1.34	0.58	0.44	G vs. F: *p* = 0.070, P_adj_=0.210; G vs. S: *p* = 0.029, P_adj_=0.087; S vs. F: *p* = 0.301, P_adj_=0.903
Total cholesterol (mmol/L)	1.27 ± 0.29 (7)	1.61 ± 0.28 (8)	1.59 ± 0.18 (8)	0.11	0.10	0.06	G vs. F: *p* = 0.039, P_adj_=0.117; G vs. S: *p* = 0.031, P_adj_=0.093; S vs. F: *p* = 0.868, P_adj_=1.000
Fructosamine (µmol/L)	488 ± 95 (7)	398 ± 38 (8)	455 ± 44 (8)	35.92	13.44	15.56	G vs. F: *p* = 0.048, P_adj_=0.144; G vs. S: *p* = 0.423, P_adj_=1.000; **S vs. F: p = 0.015**,** P**_**adj**_**=0.045ᵃ**
LDH (U/L)	1643 ± 905 (7)	1842 ± 781 (8)	1747 ± 596 (8)	342.13	276.07	210.75	G vs. F: *p* = 0.659, P_adj_=1.000; G vs. S: *p* = 0.801, P_adj_=1.000; S vs. F: *p* = 0.789, **P**_**adj**_ =1.000
Triglycerides (mmol/L)	1.44 ± 0.58 (7)	1.91 ± 0.56 (8)	1.39 ± 0.83 (8)	0.22	0.20	0.29	G vs. F: *p* = 0.136, P_adj_=0.408; G vs. S: *p* = 0.894, p__adj_=1.000; S vs. F: *p* = 0.167, P_adj_=0.501
Insulin (mg/L)	1.97 ± 0.80 (7)	2.41 ± 2.08 (8)	2.15 ± 0.99 (8)	0.30	0.74	0.35	G vs. F: *p* = 0.593, P_adj_=1.000; G vs. S: *p* = 0.703, P_adj_=1.000; S vs. F: *p* = 0.756, P_adj_=1.000
Leptin (ng/L)	2.34 ± 1.35 (7)	1.40 ± 0.36 (8)	1.87 ± 0.78 (8)	0.51	0.13	0.28	G vs. F: *p* = 0.119, P_adj_=0.357; G vs. S: *p* = 0.438, P_adj_=1.000; S vs. F: *p* = 0.153, P_adj_=0.459
Glucagon (pg/L)	2.94 ± 2.05 (7)	4.85 ± 4.09 (8)	4.68 ± 3.37 (8)	0.77	1.45	1.19	G vs. F: *p* = 0.270, P_adj_=0.810; G vs. S: *p* = 0.245, P_adj_=0.735; S vs. F: *p* = 0.929, P_adj_=1.000

### Dendritic spine density in CA1 stratum radiatum

**Fig. 4:**
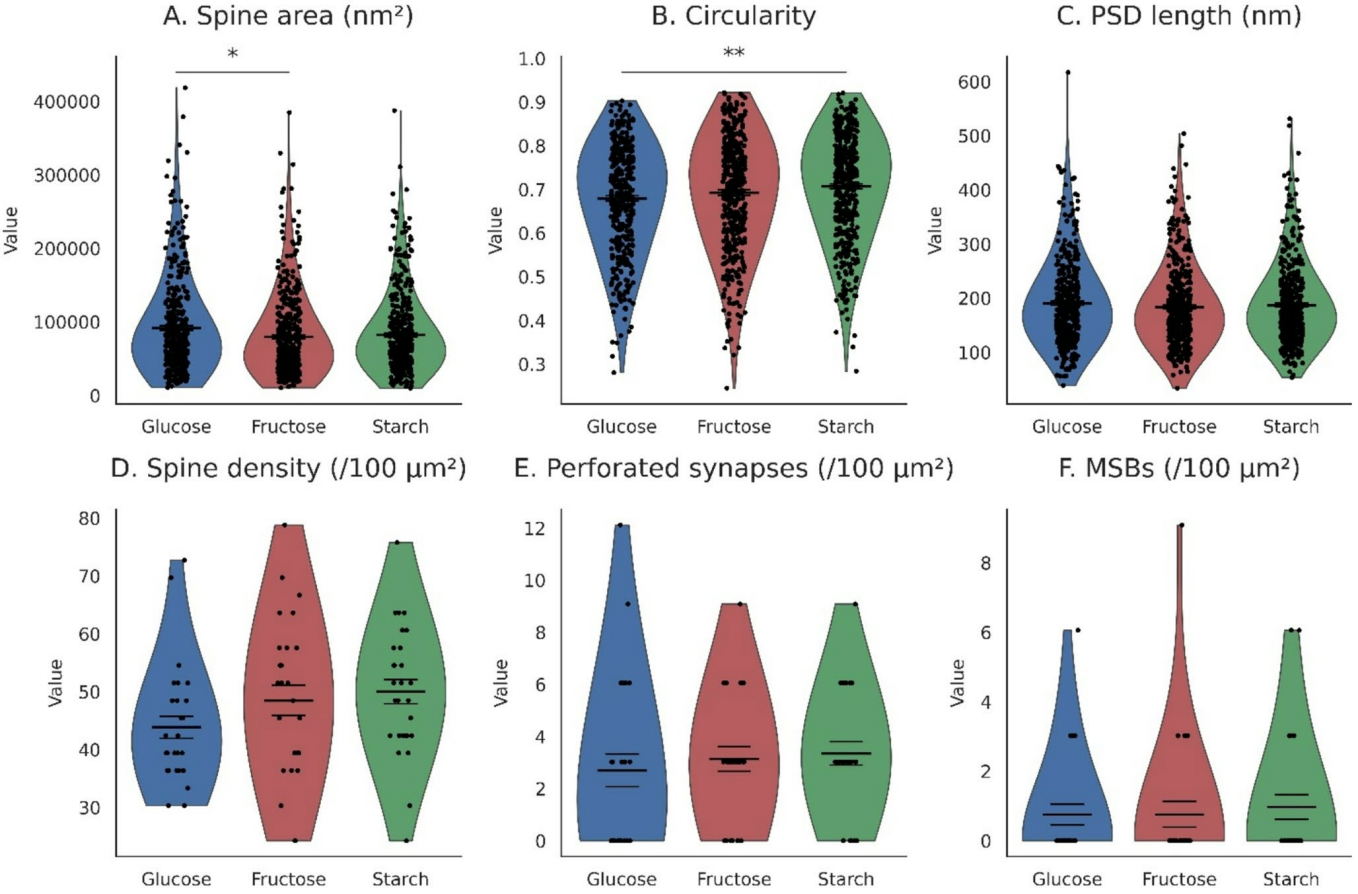
Dendritic spine and synapse ultrastructure in CA1 stratum radiatum after four weeks of glucose-, fructose-, or starch-based feeding. (A) spine area, (B) circularity, (C) PSD length, (D) spine density (per 100 μm²), (E) perforated synapse density (per 100 μm²), and (F) MSB density (per 100 μm²). Distributions are shown as violins with jittered datapoints; horizontal ticks denote mean (wide) and mean ± SE (narrow). Group comparisons used Welch’s t-tests with Bonferroni correction. Asterisks denote significant adjusted p-values (*P* < 0.05*, *P* < 0.01**). Spine morphology metrics (area, circularity, PSD length) were quantified from *n* = 404 (glucose), *n* = 462 (starch), and *n* = 448 (fructose) spines. Density measures (spines, perforated synapses, MSBs) were derived from 28 EM fields per group, each covering 33 μm² of neuropil (normalized to 100 μm²)

Quantitative electron microscopy revealed that dendritic spine density in CA1 stratum radiatum was similar across all diet groups (Fig. 4D). When normalized to 100 μm² of neuropil, spine densities were 43.83 ± 10.15 in glucose-fed rats, 48.48 ± 13.65 in fructose-fed rats, and 50.00 ± 11.02 in starch-fed controls, with no significant pairwise differences detected (Welch tests; all Bonferroni-adjusted *p* = ns). These data indicate that the overall number of dendritic spines in this hippocampal subregion was not altered by dietary carbohydrate composition.

### Spine morphology and profile area

In contrast to the stable spine densities, several spine-level morphological parameters differed among diet groups (Fig. 4A, B). Mean spine head area was reduced in fructose-fed rats (79 680 ± 56 410 nm²) compared with glucose-fed rats (91 635 ± 64 882 nm²; Bonferroni-adjusted *p* = 0.013), whereas the fructose–starch and glucose–starch comparisons were not significant. Spine perimeter also differed between diets, with glucose-fed rats showing higher values than fructose-fed rats (adjusted *p* = 0.006). In addition, circularity measurements were higher in the starch group relative to the glucose group (adjusted *p* = 0.002). Other contrasts for these metrics were nonsignificant. Together, these findings demonstrate that diet produced measurable differences in spine head geometry, particularly between glucose- and fructose-fed animals.

### Postsynaptic density (PSD) morphology

Measurements of PSD length did not differ significantly across diet groups (Fig. 4C). Mean PSD lengths were 190.62 ± 78.31 nm (glucose), 183.45 ± 75.01 nm (fructose), and 187.18 ± 73.93 nm (starch), and all pairwise comparisons were nonsignificant after Bonferroni correction (all adjusted *p* > 0.17). Likewise, the density of perforated PSDs was similar among groups (all *P* ≥ 0.406; adjusted *p* = ns; Fig. 4E), indicating no diet-related differences in the proportion of perforated synapses.

### Presynaptic structural parameters: MSBs and terminal organization

The density of multisynaptic boutons (MSBs) was comparable among all dietary groups (Fig. 4F). Glucose-, fructose-, and starch-fed animals exhibited similar MSB frequencies per 100 μm², with all Welch comparisons nonsignificant (all *P* ≥ 0.639; Bonferroni-adjusted *P* = ns). No apparent differences were observed in bouton appearance or organization.

## Discussion

In this study, we investigated whether four weeks of dietary fructose alter synaptic ultrastructure in the hippocampal CA1 region of adult rats. Across multiple structural parameters—including dendritic spine density, PSD length, perforated synapse frequency, and multisynaptic bouton (MSB) density—we found no substantial diet-dependent differences. The only detectable morphological changes were a modest reduction in dendritic spine area and an alteration in spine circularity in fructose-fed animals. These effects suggest fine-scale adjustments in spine geometry without evidence of broader synaptic remodeling or altered synaptic efficacy.

Spine area and circularity are sensitive indicators of cytoskeletal organization and spine-head contour, yet these changes were not accompanied by differences in PSD length or perforated synapse frequency—canonical ultrastructural correlates of synaptic strengthening or long-term potentiation(Rácz and Weinberg 2013). Thus, although fructose intake subtly influenced local spine geometry, it did not produce the structural constellation typically associated with sustained potentiation or depression within CA1 synapses.

At first glance, these findings may appear to contrast with earlier reports indicating that fructose exposure can significantly disturb hippocampal function. Dietary fructose has been associated with neuroinflammation, mitochondrial dysfunction, oxidative stress, and insulin resistance in the brain (Spagnuolo et al. 2020). Experimental paradigms in rodents—including prolonged fructose administration—have demonstrated impaired hippocampus-dependent learning, reduced LTP and LTD, diminished neurogenesis, and molecular signatures of synaptic dysfunction (Cisternas et al. 2015; Hsu et al. 2015; Noble et al. 2017). Parallel studies show that fructose increases inflammatory markers such as TNF-α and GFAP and reduces mitochondrial function or abundance(Cigliano et al. 2018). These biochemical and functional disturbances are frequently interpreted as evidence for compromised synaptic integrity. For example, (Cisternas et al. 2015) reported a significant *decrease* in PSD width, with no change in PSD length, together with additional synaptic alterations after eight weeks of high-fructose exposure delivered in drinking water. This duration and delivery method impose a substantially greater metabolic load than the four-week, isocaloric solid-diet regimen used here. Their findings suggest that PSD remodeling may emerge primarily under prolonged or metabolically stressful fructose exposure, conditions not present in the current design.

However, several key differences between these studies and the present work help explain the divergence in outcomes. Many prior studies delivering fructose in drinking water substantially increase voluntary intake and metabolic stress, thereby exaggerating neurobiological effects compared to isocaloric solid-diet paradigms. Moreover, a consistent literature shows that *adolescent animals* are markedly more vulnerable to diet-induced hippocampal alterations, whereas adults display milder or transient effects (Cisternas et al. 2015; Hsu et al. 2015; Noble et al. 2017) Early-life or maternal fructose exposure also induces long-lasting changes in synaptic proteins, transcriptomic signatures, and epigenetic regulation—such as BDNF promoter hypermethylation (Kageyama et al. 2022; Zou et al. 2022, 2023)—none of which directly apply to the adult-only design of the present study.

Importantly, outcomes in adult and aging animals are heterogeneous. Chronic 11% HFCS consumption in aged rats did not adversely affect learning, memory, or hippocampal or total brain volumes (Çevreli et al. 2025), reinforcing the notion that mature hippocampal circuits may be relatively resilient to moderate fructose exposure in the absence of major metabolic burden. Diet composition further modulates outcomes: high-fat–fructose paradigms can impair hippocampal synaptic plasticity and alter serum lipid profiles (Micháliková et al. 2018)—a pattern not observed under the controlled macronutrient conditions used here.

The present study employed adult rats fed isocaloric AIN-93G solid diets with controlled carbohydrate substitution, minimizing the metabolic overload typical of liquid-fructose designs. Under these controlled conditions, systemic metabolic parameters remained broadly stable, likely limiting downstream effects on neuronal and synaptic structure. This aligns with evidence that moderate, diet-controlled fructose exposure in adults often yields limited neurostructural or cognitive disruption (Çevreli et al. 2025) These contrasts highlight that metabolic stress appears to be a key determinant of fructose-related neural outcomes; in its absence, as in the present isocaloric design, many of the hippocampal alterations reported in prior work are not expected to manifest.

An additional discrepancy with several fructose-feeding studies is the absence of elevated fructosamine, which is typically increased under high-fructose or high-glycemic feeding regimes. In the present work, fructosamine was *lower* in fructose-fed animals compared to starch controls. This is likely attributable to the isocaloric solid-diet formulation in a regulated intake (fructose supplied as 65% dietary carbohydrate rather than as a liquid solution that promotes overconsumption), and relatively stable glycemia, since fructosamine reflects average circulating glucose over the preceding 2–3 weeks. Many studies reporting elevated fructosamine rely on liquid fructose paradigms that produce higher glycemic and hepatic fructolysis load. Our solid, isocaloric AIN-93G design avoids the overconsumption and rapid hepatic fructose delivery typical of liquid sugar paradigms, which likely explains the lower fructosamine in our fructose group (Gallagher et al. 2016; Sundborn et al. 2019; Bouwman et al. 2020; Fisher et al. 2024) Taken together, the lower fructosamine values observed here are consistent with a metabolically stable environment, which aligns with the absence of broad ultrastructural differences among diet groups.

A limitation of the present study is that food intake and caloric consumption were not quantified. Although all groups were maintained on isocaloric AIN-93G-based diets with identical macronutrient distributions except for the carbohydrate source, the absence of intake measurements precludes evaluation of subtle group-level differences in voluntary consumption. This constraint should be considered when comparing our metabolic findings with studies using measured caloric intake or liquid fructose paradigms, which often induce higher total sugar consumption. Nonetheless, the stability of circulating biochemical parameters across groups suggests that major differences in energy intake were unlikely under the controlled solid-diet conditions used here.

It is also plausible that fructose’s neural effects occur predominantly upstream of overt ultrastructure. Fructose can perturb mitochondrial function, redox balance, and inflammatory pathways (Cigliano et al. 2018), and microglia—via the fructose transporter GLUT5—may undergo fructose-driven metabolic reprogramming capable of influencing neuronal activity without directly altering excitatory synapse architecture (Ting 2024). These glial-centric mechanisms could contribute to functional modulation in the absence of detectable changes in dendritic spines or PSD morphology. The role of GLUT5 in hippocampal fructose handling also warrant clarification. Although fructose transport into the brain is generally attributed to GLUT5, its precise regional and cellular distribution remains unresolved; notably, Oppelt et al. (2017) did not detect GLUT5 in hippocampal neurons in their study. Thus, potential microglial or neuronal fructose metabolism in CA1 should be interpreted with caution. Importantly, we did not assess microglial activation, microglial metabolism, or GLUT5 expression in the present study; therefore, these pathways are discussed solely as contextual frameworks derived from existing literature and not as conclusions supported by our data.

Taken together, our findings indicate that adult CA1 synapses remain structurally stable under short-term, diet-controlled fructose intake. However, the subtle alterations in spine micro-geometry observed here may represent early or compensatory morphological signatures of diet-related plasticity that do not progress to canonical synaptic reorganization under stable metabolic conditions. Future studies should assess glial physiology, mitochondrial bioenergetics, microglial metabolism, and electrophysiological plasticity, and systematically vary age, dietary background, exposure duration, and delivery method to determine thresholds at which fructose transitions from producing fine-scale spine changes to more pronounced structural or functional impairments.

Overall, short-term fructose exposure in adults produced modest adjustments in dendritic spine geometry without altering the broader ultrastructural landscape of CA1 synapses. These results refine our understanding of how dietary sugars influence hippocampal circuitry and suggest that, under moderate and metabolically controlled conditions, synaptic degradation is unlikely to be the primary driver of fructose-related cognitive changes in adults.

## Supplementary Information

Below is the link to the electronic supplementary material.

Supplementary Material 1

## Data Availability

All datasets generated and analysed during the current study are openly available in the Zenodo repository at the following DOI: https://doi.org/10.5281/zenodo.18338177.

## References

[CR1] Alam YH, Kim R, Jang C (2022) Metabolism and Health Impacts of Dietary Sugars. J Lipid Atheroscler 11:20–38. 10.12997/jla.2022.11.1.20 35118020 10.12997/jla.2022.11.1.20 PMC8792817

[CR2] Babits R, Szőke B, Sótonyi P, Rácz B (2016) Food restriction modifies ultrastructure of hippocampal synapses. Hippocampus 26:437–444. 10.1002/hipo.22533 26386363 10.1002/hipo.22533

[CR3] Bourne JN, Harris KM (2008) Balancing structure and function at hippocampal dendritic spines. Annu Rev Neurosci 31:47–67. 10.1146/annurev.neuro.31.060407.125646 18284372 10.1146/annurev.neuro.31.060407.125646 PMC2561948

[CR4] Bouwman LMS, Nieuwenhuizen AG, Swarts HJM et al (2020) Metabolic effects of the dietary monosaccharides fructose, fructose-glucose, or glucose in mice fed a starch-containing moderate high-fat diet. Physiol Rep 8:e14350. 10.14814/phy2.14350 10.14814/phy2.14350 PMC7002529 32026655

[CR5] Çevreli B, Öztürk G, Baygül O (2025) Cognitive and neuroanatomical effects of chronic high-fructose corn syrup consumption in the aging rat brain. BMC Neuroscience 2025 26:1-26:66. 10.1186/s12868-025-00984-2 10.1186/s12868-025-00984-2 PMC12664247 41315901

[CR6] Cigliano L, Spagnuolo MS, Crescenzo R et al (2018) Short-Term Fructose Feeding Induces Inflammation and Oxidative Stress in the Hippocampus of Young and Adult Rats. Mol Neurobiol 55:2869–2883. 10.1007/s12035-017-0518-2 28455700 10.1007/s12035-017-0518-2

[CR7] Cisternas P, Salazar P, Serrano FG et al (2015) Fructose consumption reduces hippocampal synaptic plasticity underlying cognitive performance. BBA - Mol Basis Disease 1852:2379–2390. ttps://doi.org/10.1016/j.bbadis.2015.08.016 10.1016/j.bbadis.2015.08.016 PMC5369608 26300486

[CR8] Fisher SL, Campbell GJ, Senior A, Bell-Anderson K (2024) The effect of high-sugar feeding on rodent metabolic phenotype: a systematic review and meta-analysis. npj metabolic health disease 2(40):1-13. 10.1038/s44324-024-00043-0 10.1038/s44324-024-00043-0 PMC12118738 40604308

[CR9] Gallagher C, Keogh JB, Pedersen E, Clifton PM (2016) Fructose acute effects on glucose, insulin, and triglyceride after a solid meal compared with sucralose and sucrose in a randomized crossover study. Am J Clin Nutr 103:1453–1457. 10.3945/ajcn.115.129866 27099245 10.3945/ajcn.115.129866

[CR10] Harris KM, Stevens JK (1989) Dendritic spines of CA1 pyramidal cells in the rat hippocampus: serial electron microscopy with reference to their biophysical characteristics. J Neurosci 9:2982–2997. 10.1523/JNEUROSCI.09-08-02982.1989 2769375 10.1523/JNEUROSCI.09-08-02982.1989 PMC6569708

[CR11] Harris KM, Weinberg R (2012) Ultrastructure of Synapses in the Mammalian Brain. Cold Spring Harb Perspect Biol 4:a005587. 10.1101/cshperspect.a005587 10.1101/cshperspect.a005587 PMC3331701 22357909

[CR12] Hsu TM, Konanur VR, Taing L et al (2015) Effects of sucrose and high fructose corn syrup consumption on spatial memory function and hippocampal neuroinflammation in adolescent rats. Hippocampus 25:227–239. 10.1002/hipo.22368 25242636 10.1002/hipo.22368

[CR13] Huang Y, Chen Z, Chen B et al (2023) Dietary sugar consumption and health: umbrella review. BMJ 2023;381:e071609 pp. 1-18 10.1136/bmj-2022-071609 10.1136/bmj-2022-071609 PMC10074550 37019448

[CR14] Kageyama I, Yamada H, Munetsuna E et al (2022) Differential effects of excess high-fructose corn syrup on DNA methylation of hippocampal neurotrophic factor in childhood and adolescence. PLoS ONE 17:e0270144. 10.1371/journal.pone.0270144 35714129 10.1371/journal.pone.0270144 PMC9205497

[CR15] Ma X, Nan F, Liang H et al (2022) Excessive intake of sugar: An accomplice of inflammation. Front Immunol 13:988481. 10.3389/fimmu.2022.988481 36119103 10.3389/fimmu.2022.988481 PMC9471313

[CR16] Micháliková D, Kaprinay BT, Lipták B et al (2018) Effect of high-fat-fructose diet on synaptic plasticity in hippocampus and lipid profile of blood serum of rat: pharmacological possibilities of affecting risk factors. Eur Pharm J 65:12–16. 10.2478/afpuc-2018-0008

[CR17] Noble EE, Hsu TM, Liang J, Kanoski SE (2017) Early-life sugar consumption has long-term negative effects on memory function in male rats. Nutr Neurosci. 22(4):273-283 10.1080/1028415X.2017.1378851 10.1080/1028415X.2017.1378851 PMC5972076 28944721

[CR18] Oppelt SA, Zhang W, Tolan DR (2017) Specific regions of the brain are capable of fructose metabolism. Brain Res 1657:312–322. 10.1016/j.brainres.2016.12.022 28034722 10.1016/j.brainres.2016.12.022 PMC5420427

[CR19] Rácz B, Weinberg RJ (2013) Microdomains in forebrain spines: an ultrastructural perspective. Mol Neurobiol 47:77–89. 10.1007/s12035-012-8345-y 22983912 10.1007/s12035-012-8345-y PMC3538892

[CR20] Reeves PG, Nielsen FH, Fahey GC (1993) AIN-93 purified diets for laboratory rodents: Final report of the American Institute of Nutrition Ad Hoc Writing Committee on the Reformulation of the AIN-76A rodent diet. J Nutr 123:1939–1951. 10.1093/jn/123.11.1939 8229312 10.1093/jn/123.11.1939

[CR21] Sorra KE, Harris KM (1993) Occurrence and three-dimensional structure of multiple synapses between individual radiatum axons and CA1 pyramidal cells. J Neurosci 13:3736–3748. 10.1523/JNEUROSCI.13-09-03736.1993 8366344 10.1523/JNEUROSCI.13-09-03736.1993 PMC6576466

[CR22] Spagnuolo MS, Iossa S, Cigliano L (2020) Sweet but Bitter: Focus on Fructose Impact on Brain Function in Rodent Models. Nutrients 13:E1–E1. 10.3390/nu13010001 10.3390/nu13010001 PMC7821920 33374894

[CR23] Sundborn G, Thornley S, Merriman TR et al (2019) Are Liquid Sugars Different from Solid Sugar in Their Ability to Cause Metabolic Syndrome? Obes (Silver Spring) 27:879–887. 10.1002/oby.22472 10.1002/oby.22472 31054268

[CR24] Ting KKY (2024) Fructose overconsumption-induced reprogramming of microglia metabolism and function. Front Immunol 15:1375453. 10.3389/fimmu.2024.1375453 38596671 10.3389/fimmu.2024.1375453 PMC11002174

[CR25] Toni N, Buchs P-A, Nikonenko I et al (1999) LTP promotes formation of multiple spine synapses between a single axon terminal and a dendrite. Nature 402:421–425. 10.1038/46574 10586883 10.1038/46574

[CR27] Zou Y, Guo Q, Chang Y et al (2022) Learning and memory impairment and transcriptomic profile in hippocampus of offspring after maternal fructose exposure during gestation and lactation. Food Chem Toxicol 169:113394. 10.1016/j.fct.2022.113394 36049592 10.1016/j.fct.2022.113394

[CR26] Zou Y, Guo Q, Chang Y et al (2023) Alternative splicing affects synapses in the hippocampus of offspring after maternal fructose exposure during gestation and lactation. Chem Biol Interact 379:110518. 10.1016/j.cbi.2023.110518 37121297 10.1016/j.cbi.2023.110518

