# Supplementary material for: Selective alterations in CA1 spine morphology following dietary fructose intake
Source: Brain Struct Funct. 2026 May 5;231(5):59. doi: 10.1007/s00429-026-03102-y (PMC13139255; doi:10.1007/s00429-026-03102-y)
Supplement: Supplementary file 1 — Supplementary Material 1 [file 429_2026_3102_MOESM1_ESM.pdf]

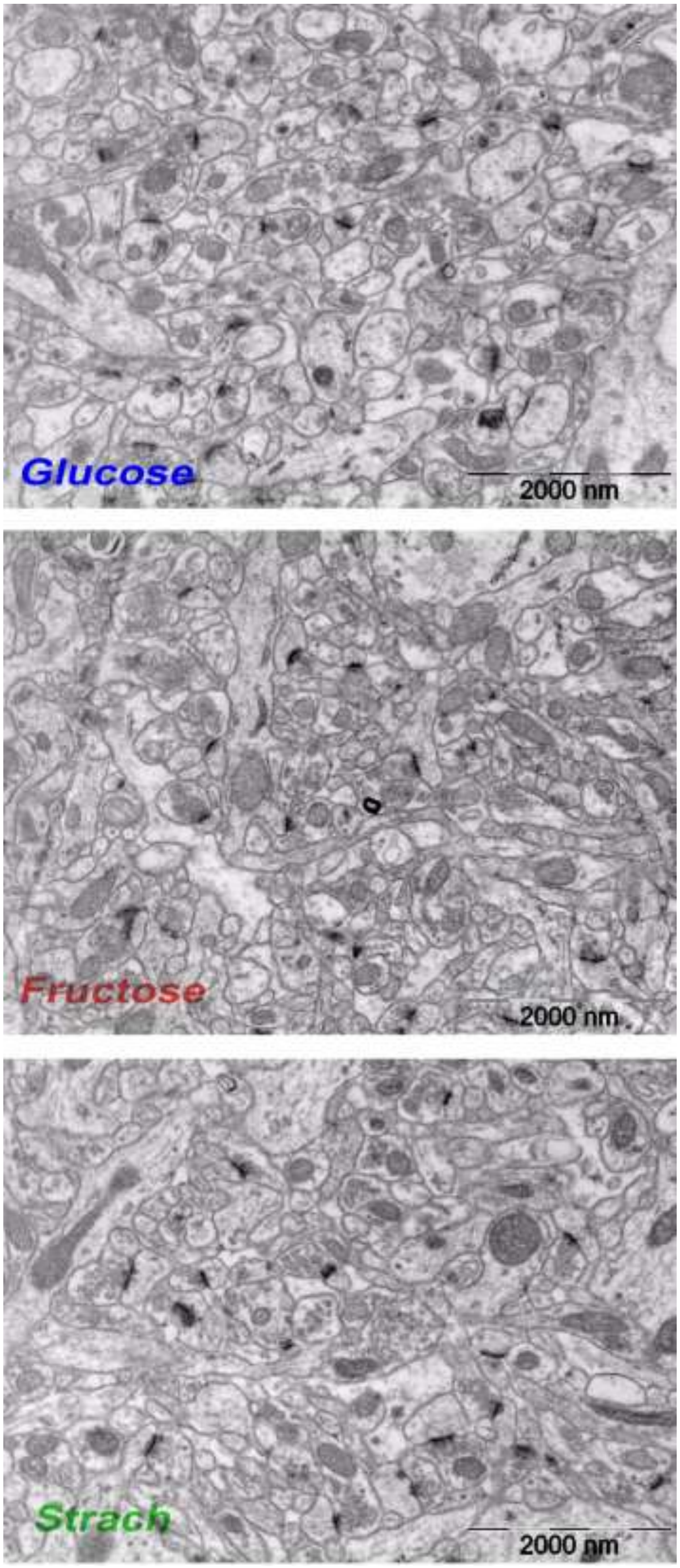

Supplementary Figure 1. Representative electron micrographs from each diet group. Representative transmission electron micrographs of CA1 stratum radiatum from rats fed glucose, fructose, or starch diets for four weeks. Images illustrate general neuropil organization and typical synaptic elements. These examples are provided for reference; the subtle ultrastructural differences reported in the study were identified through quantitative analysis. Scale bar: 2000 nm.
